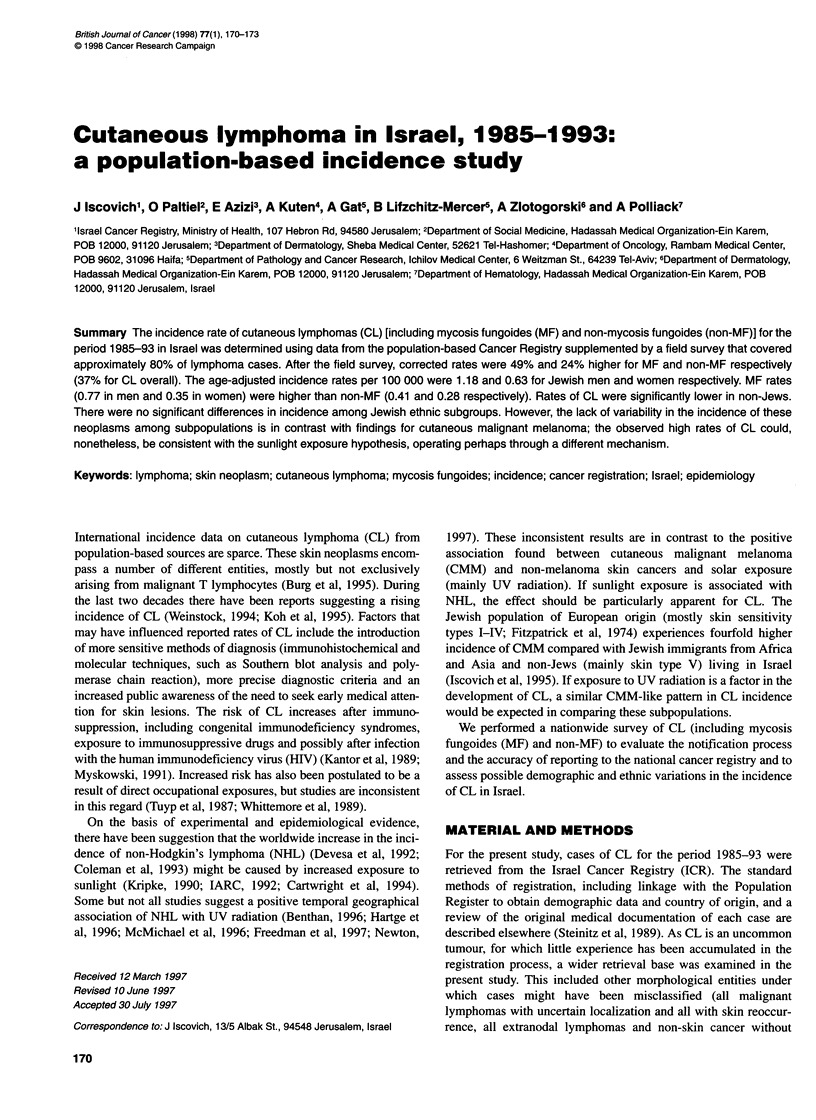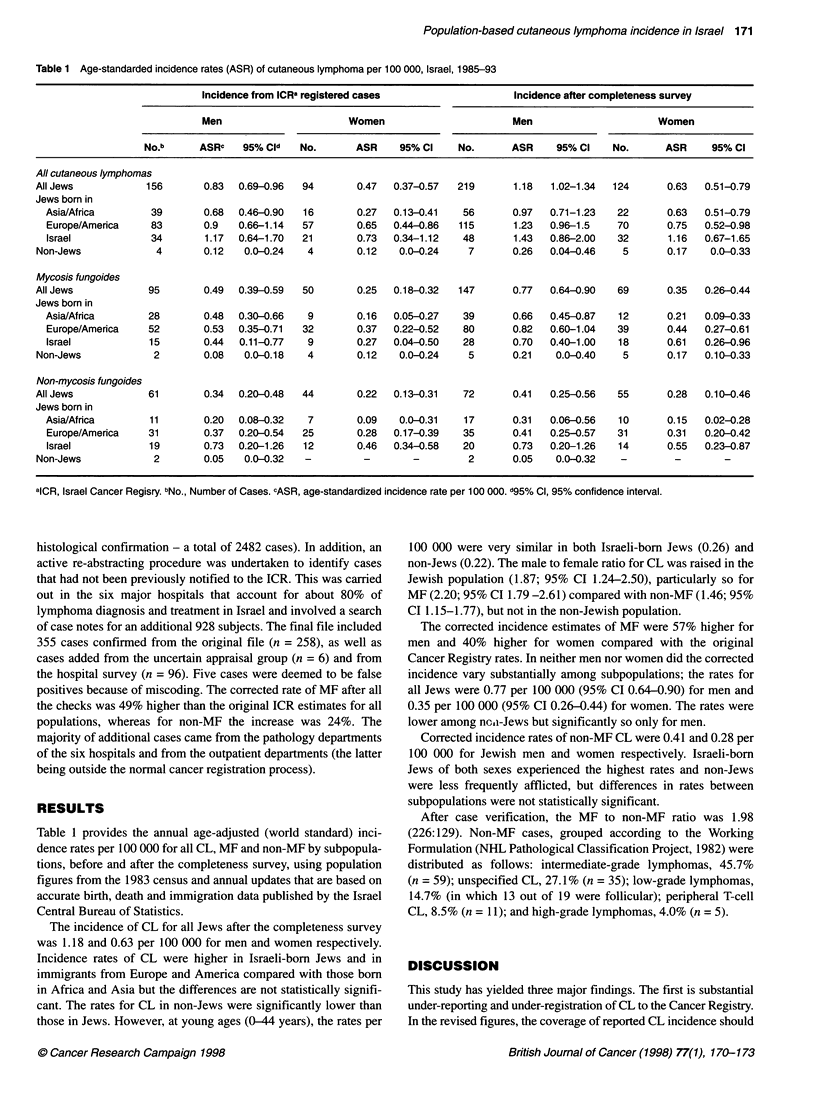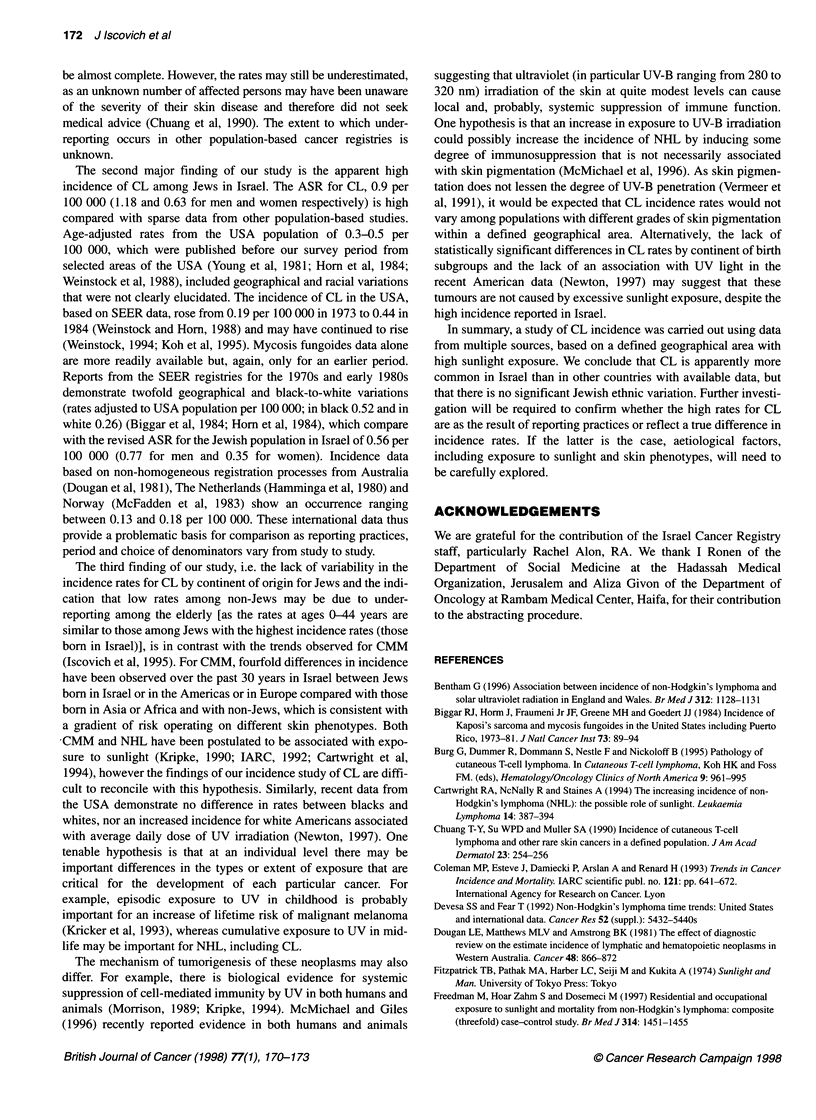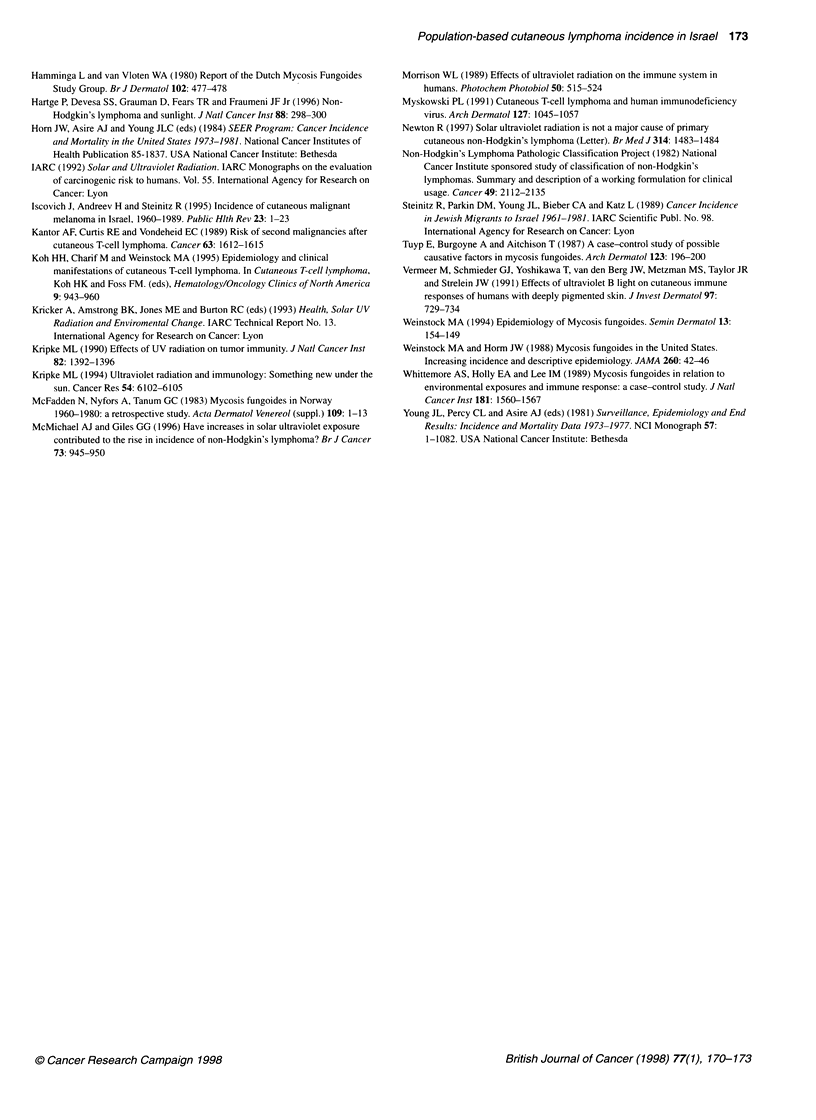# Cutaneous lymphoma in Israel, 1985-1993: a population-based incidence study.

**DOI:** 10.1038/bjc.1998.27

**Published:** 1998

**Authors:** J. Iscovich, O. Paltiel, E. Azizi, A. Kuten, A. Gat, B. Lifzchitz-Mercer, A. Zlotogorski, A. Polliack

**Affiliations:** Israel Cancer Registry, Ministry of Health, Jerusalem.

## Abstract

The incidence rate of cutaneous lymphomas (CL) [including mycosis fungoides (MF) and non-mycosis fungoides (non-MF)] for the period 1985-93 in Israel was determined using data from the population-based Cancer Registry supplemented by a field survey that covered approximately 80% of lymphoma cases. After the field survey, corrected rates were 49% and 24% higher for MF and non-MF respectively (37% for CL overall). The age-adjusted incidence rates per 100,000 were 1.18 and 0.63 for Jewish men and women respectively. MF rates (0.77 in men and 0.35 in women) were higher than non-MF (0.41 and 0.28 respectively). Rates of CL were significantly lower in non-Jews. There were no significant differences in incidence among Jewish ethnic subgroups. However, the lack of variability in the incidence of these neoplasms among subpopulations is in contrast with findings for cutaneous malignant melanoma; the observed high rates of CL could, nonetheless, be consistent with the sunlight exposure hypothesis, operating perhaps through a different mechanism.


British Journal of Cancer (1998) 77(1), 170–173
© 1998 Cancer Research Campaign

### Cutaneous lymphoma in Israel, 1985–1993: a population-based incidence study

**J Iscovich[1], O Paltiel[2], E Azizi[3], A Kuten[4], A Gat[5], B Lifzchitz-Mercer[5], A Zlotogorski[6] and A Polliack[7]**

[1]Israel Cancer Registry, Ministry of Health, 107 Hebron Rd, 94580 Jerusalem; [2]Department of Social Medicine, Hadassah Medical Organization-Ein Karem, POB 12000, 91120 Jerusalem; [3]Department of Dermatology, Sheba Medical Center, 52621 Tel-Hashomer; [4]Department of Oncology, Rambam Medical Center, POB 9602, 31096 Haifa; [5]Department of Pathology and Cancer Research, Ichilov Medical Center, 6 Weitzman St., 64239 Tel-Aviv; [6]Department of Dermatology, Hadassah Medical Organization-Ein Karem, POB 12000, 91120 Jerusalem; [7]Department of Hematology, Hadassah Medical Organization-Ein Karem, POB 12000, 91120 Jerusalem, Israel

**Summary** The incidence rate of cutaneous lymphomas (CL) [including mycosis fungoides (MF) and non-mycosis fungoides (non-MF)] for the period 1985–93 in Israel was determined using data from the population-based Cancer Registry supplemented by a field survey that covered approximately 80% of lymphoma cases. After the field survey, corrected rates were 49% and 24% higher for MF and non-MF respectively (37% for CL overall). The age-adjusted incidence rates per 100 000 were 1.18 and 0.63 for Jewish men and women respectively. MF rates (0.77 in men and 0.35 in women) were higher than non-MF (0.41 and 0.28 respectively). Rates of CL were significantly lower in non-Jews. There were no significant differences in incidence among Jewish ethnic subgroups. However, the lack of variability in the incidence of these neoplasms among subpopulations is in contrast with findings for cutaneous malignant melanoma; the observed high rates of CL could, nonetheless, be consistent with the sunlight exposure hypothesis, operating perhaps through a different mechanism.

**Keywords**: lymphoma; skin neoplasm; cutaneous lymphoma; mycosis fungoides; incidence; cancer registration; Israel; epidemiology

International incidence data on cutaneous lymphoma (CL) from population-based sources are sparce. These skin neoplasms encompass a number of different entities, mostly but not exclusively arising from malignant T lymphocytes (Burg et al, 1995). During the last two decades there have been reports suggesting a rising incidence of CL (Weinstock, 1994; Koh et al, 1995). Factors that may have influenced reported rates of CL include the introduction of more sensitive methods of diagnosis (immunohistochemical and molecular techniques, such as Southern blot analysis and polymerase chain reaction), more precise diagnostic criteria and an increased public awareness of the need to seek early medical attention for skin lesions. The risk of CL increases after immunosuppression, including congenital immunodeficiency syndromes, exposure to immunosuppressive drugs and possibly after infection with the human immunodeficiency virus (HIV) (Kantor et al, 1989; Myskowski, 1991). Increased risk has also been postulated to be a result of direct occupational exposures, but studies are inconsistent in this regard (Tuyp et al, 1987; Whittemore et al, 1989).

On the basis of experimental and epidemiological evidence, there have been suggestion that the worldwide increase in the incidence of non-Hodgkin's lymphoma (NHL) (Devesa et al, 1992; Coleman et al, 1993) might be caused by increased exposure to sunlight (Kripke, 1990; IARC, 1992; Cartwright et al, 1994). Some but not all studies suggest a positive temporal geographical association of NHL with UV radiation (Benthan, 1996; Hartge et al, 1996; McMichael et al, 1996; Freedman et al, 1997; Newton, 1997). These inconsistent results are in contrast to the positive association found between cutaneous malignant melanoma (CMM) and non-melanoma skin cancers and solar exposure (mainly UV radiation). If sunlight exposure is associated with NHL, the effect should be particularly apparent for CL. The Jewish population of European origin (mostly skin sensitivity types I–IV; Fitzpatrick et al, 1974) experiences fourfold higher incidence of CMM compared with Jewish immigrants from Africa and Asia and non-Jews (mainly skin type V) living in Israel (Iscovich et al, 1995). If exposure to UV radiation is a factor in the development of CL, a similar CMM-like pattern in CL incidence would be expected in comparing these subpopulations.

We performed a nationwide survey of CL (including mycosis fungoides (MF) and non-MF) to evaluate the notification process and the accuracy of reporting to the national cancer registry and to assess possible demographic and ethnic variations in the incidence of CL in Israel.

### MATERIAL AND METHODS

For the present study, cases of CL for the period 1985–93 were retrieved from the Israel Cancer Registry (ICR). The standard methods of registration, including linkage with the Population Register to obtain demographic data and country of origin, and a review of the original medical documentation of each case are described elsewhere (Steinitz et al, 1989). As CL is an uncommon tumour, for which little experience has been accumulated in the registration process, a wider retrieval base was examined in the present study. This included other morphological entities under which cases might have been misclassified (all malignant lymphomas with uncertain localization and all with skin reoccurrence, all extranodal lymphomas and non-skin cancer without

*Received 12 March 1997*
*Revised 10 June 1997*
*Accepted 30 July 1997*

*Correspondence to:* J Iscovich, 13/5 Albak St., 94548 Jerusalem, Israel

**Table 1** Age-standarded incidence rates (ASR) of cutaneous lymphoma per 100 000, Israel, 1985–93

| | Incidence from ICR[a] registered cases | | | | | | Incidence after completeness survey | | | | | |
|---|---|---|---|---|---|---|---|---|---|---|---|---|
| | Men | | | Women | | | Men | | | Women | | |
| | No.[b] | ASR[c] | 95% CI[d] | No. | ASR | 95% CI | No. | ASR | 95% CI | No. | ASR | 95% CI |
| *All cutaneous lymphomas* | | | | | | | | | | | | |
| All Jews | 156 | 0.83 | 0.69–0.96 | 94 | 0.47 | 0.37–0.57 | 219 | 1.18 | 1.02–1.34 | 124 | 0.63 | 0.51–0.79 |
| Jews born in | | | | | | | | | | | | |
| Asia/Africa | 39 | 0.68 | 0.46–0.90 | 16 | 0.27 | 0.13–0.41 | 56 | 0.97 | 0.71–1.23 | 22 | 0.63 | 0.51–0.79 |
| Europe/America | 83 | 0.9 | 0.66–1.14 | 57 | 0.65 | 0.44–0.86 | 115 | 1.23 | 0.96–1.5 | 70 | 0.75 | 0.52–0.98 |
| Israel | 34 | 1.17 | 0.64–1.70 | 21 | 0.73 | 0.34–1.12 | 48 | 1.43 | 0.86–2.00 | 32 | 1.16 | 0.67–1.65 |
| Non-Jews | 4 | 0.12 | 0.0–0.24 | 4 | 0.12 | 0.0–0.24 | 7 | 0.26 | 0.04–0.46 | 5 | 0.17 | 0.0–0.33 |
| *Mycosis fungoides* | | | | | | | | | | | | |
| All Jews | 95 | 0.49 | 0.39–0.59 | 50 | 0.25 | 0.18–0.32 | 147 | 0.77 | 0.64–0.90 | 69 | 0.35 | 0.26–0.44 |
| Jews born in | | | | | | | | | | | | |
| Asia/Africa | 28 | 0.48 | 0.30–0.66 | 9 | 0.16 | 0.05–0.27 | 39 | 0.66 | 0.45–0.87 | 12 | 0.21 | 0.09–0.33 |
| Europe/America | 52 | 0.53 | 0.35–0.71 | 32 | 0.37 | 0.22–0.52 | 80 | 0.82 | 0.60–1.04 | 39 | 0.44 | 0.27–0.61 |
| Israel | 15 | 0.44 | 0.11–0.77 | 9 | 0.27 | 0.04–0.50 | 28 | 0.70 | 0.40–1.00 | 18 | 0.61 | 0.26–0.96 |
| Non-Jews | 2 | 0.08 | 0.0–0.18 | 4 | 0.12 | 0.0–0.24 | 5 | 0.21 | 0.0–0.40 | 5 | 0.17 | 0.10–0.33 |
| *Non-mycosis fungoides* | | | | | | | | | | | | |
| All Jews | 61 | 0.34 | 0.20–0.48 | 44 | 0.22 | 0.13–0.31 | 72 | 0.41 | 0.25–0.56 | 55 | 0.28 | 0.10–0.46 |
| Jews born in | | | | | | | | | | | | |
| Asia/Africa | 11 | 0.20 | 0.08–0.32 | 7 | 0.09 | 0.0–0.31 | 17 | 0.31 | 0.06–0.56 | 10 | 0.15 | 0.02–0.28 |
| Europe/America | 31 | 0.37 | 0.20–0.54 | 25 | 0.28 | 0.17–0.39 | 35 | 0.41 | 0.25–0.57 | 31 | 0.31 | 0.20–0.42 |
| Israel | 19 | 0.73 | 0.20–1.26 | 12 | 0.46 | 0.34–0.58 | 20 | 0.73 | 0.20–1.26 | 14 | 0.55 | 0.23–0.87 |
| Non-Jews | 2 | 0.05 | 0.0–0.32 | – | – | – | 2 | 0.05 | 0.0–0.32 | – | – | – |

[a]ICR, Israel Cancer Regisry. [b]No., Number of Cases. [c]ASR, age-standardized incidence rate per 100 000. [d]95% CI, 95% confidence interval.

histological confirmation – a total of 2482 cases). In addition, an active re-abstracting procedure was undertaken to identify cases that had not been previously notified to the ICR. This was carried out in the six major hospitals that account for about 80% of lymphoma diagnosis and treatment in Israel and involved a search of case notes for an additional 928 subjects. The final file included 355 cases confirmed from the original file ($n$ = 258), as well as cases added from the uncertain appraisal group ($n$ = 6) and from the hospital survey ($n$ = 96). Five cases were deemed to be false positives because of miscoding. The corrected rate of MF after all the checks was 49% higher than the original ICR estimates for all populations, whereas for non-MF the increase was 24%. The majority of additional cases came from the pathology departments of the six hospitals and from the outpatient departments (the latter being outside the normal cancer registration process).

### RESULTS

Table 1 provides the annual age-adjusted (world standard) incidence rates per 100 000 for all CL, MF and non-MF by subpopulations, before and after the completeness survey, using population figures from the 1983 census and annual updates that are based on accurate birth, death and immigration data published by the Israel Central Bureau of Statistics.

The incidence of CL for all Jews after the completeness survey was 1.18 and 0.63 per 100 000 for men and women respectively. Incidence rates of CL were higher in Israeli-born Jews and in immigrants from Europe and America compared with those born in Africa and Asia but the differences are not statistically significant. The rates for CL in non-Jews were significantly lower than those in Jews. However, at young ages (0–44 years), the rates per 100 000 were very similar in both Israeli-born Jews (0.26) and non-Jews (0.22). The male to female ratio for CL was raised in the Jewish population (1.87; 95% CI 1.24–2.50), particularly so for MF (2.20; 95% CI 1.79 –2.61) compared with non-MF (1.46; 95% CI 1.15–1.77), but not in the non-Jewish population.

The corrected incidence estimates of MF were 57% higher for men and 40% higher for women compared with the original Cancer Registry rates. In neither men nor women did the corrected incidence vary substantially among subpopulations; the rates for all Jews were 0.77 per 100 000 (95% CI 0.64–0.90) for men and 0.35 per 100 000 (95% CI 0.26–0.44) for women. The rates were lower among non-Jews but significantly so only for men.

Corrected incidence rates of non-MF CL were 0.41 and 0.28 per 100 000 for Jewish men and women respectively. Israeli-born Jews of both sexes experienced the highest rates and non-Jews were less frequently afflicted, but differences in rates between subpopulations were not statistically significant.

After case verification, the MF to non-MF ratio was 1.98 (226:129). Non-MF cases, grouped according to the Working Formulation (NHL Pathological Classification Project, 1982) were distributed as follows: intermediate-grade lymphomas, 45.7% ($n$ = 59); unspecified CL, 27.1% ($n$ = 35); low-grade lymphomas, 14.7% (in which 13 out of 19 were follicular); peripheral T-cell CL, 8.5% ($n$ = 11); and high-grade lymphomas, 4.0% ($n$ = 5).

### DISCUSSION

This study has yielded three major findings. The first is substantial under-reporting and under-registration of CL to the Cancer Registry. In the revised figures, the coverage of reported CL incidence should be almost complete. However, the rates may still be underestimated, as an unknown number of affected persons may have been unaware of the severity of their skin disease and therefore did not seek medical advice (Chuang et al, 1990). The extent to which under-reporting occurs in other population-based cancer registries is unknown.

The second major finding of our study is the apparent high incidence of CL among Jews in Israel. The ASR for CL, 0.9 per 100 000 (1.18 and 0.63 for men and women respectively) is high compared with sparse data from other population-based studies. Age-adjusted rates from the USA population of 0.3–0.5 per 100 000, which were published before our survey period from selected areas of the USA (Young et al, 1981; Horn et al, 1984; Weinstock et al, 1988), included geographical and racial variations that were not clearly elucidated. The incidence of CL in the USA, based on SEER data, rose from 0.19 per 100 000 in 1973 to 0.44 in 1984 (Weinstock and Horn, 1988) and may have continued to rise (Weinstock, 1994; Koh et al, 1995). Mycosis fungoides data alone are more readily available but, again, only for an earlier period. Reports from the SEER registries for the 1970s and early 1980s demonstrate twofold geographical and black-to-white variations (rates adjusted to USA population per 100 000; in black 0.52 and in white 0.26) (Biggar et al, 1984; Horn et al, 1984), which compare with the revised ASR for the Jewish population in Israel of 0.56 per 100 000 (0.77 for men and 0.35 for women). Incidence data based on non-homogeneous registration processes from Australia (Dougan et al, 1981), The Netherlands (Hamminga et al, 1980) and Norway (McFadden et al, 1983) show an occurrence ranging between 0.13 and 0.18 per 100 000. These international data thus provide a problematic basis for comparison as reporting practices, period and choice of denominators vary from study to study.

The third finding of our study, i.e. the lack of variability in the incidence rates for CL by continent of origin for Jews and the indication that low rates among non-Jews may be due to under-reporting among the elderly [as the rates at ages 0–44 years are similar to those among Jews with the highest incidence rates (those born in Israel)], is in contrast with the trends observed for CMM (Iscovich et al, 1995). For CMM, fourfold differences in incidence have been observed over the past 30 years in Israel between Jews born in Israel or in the Americas or in Europe compared with those born in Asia or Africa and with non-Jews, which is consistent with a gradient of risk operating on different skin phenotypes. Both CMM and NHL have been postulated to be associated with exposure to sunlight (Kripke, 1990; IARC, 1992; Cartwright et al, 1994), however the findings of our incidence study of CL are difficult to reconcile with this hypothesis. Similarly, recent data from the USA demonstrate no difference in rates between blacks and whites, nor an increased incidence for white Americans associated with average daily dose of UV irradiation (Newton, 1997). One tenable hypothesis is that at an individual level there may be important differences in the types or extent of exposure that are critical for the development of each particular cancer. For example, episodic exposure to UV in childhood is probably important for an increase of lifetime risk of malignant melanoma (Kricker et al, 1993), whereas cumulative exposure to UV in mid-life may be important for NHL, including CL.

The mechanism of tumorigenesis of these neoplasms may also differ. For example, there is biological evidence for systemic suppression of cell-mediated immunity by UV in both humans and animals (Morrison, 1989; Kripke, 1994). McMichael and Giles (1996) recently reported evidence in both humans and animals suggesting that ultraviolet (in particular UV-B ranging from 280 to 320 nm) irradiation of the skin at quite modest levels can cause local and, probably, systemic suppression of immune function. One hypothesis is that an increase in exposure to UV-B irradiation could possibly increase the incidence of NHL by inducing some degree of immunosuppression that is not necessarily associated with skin pigmentation (McMichael et al, 1996). As skin pigmentation does not lessen the degree of UV-B penetration (Vermeer et al, 1991), it would be expected that CL incidence rates would not vary among populations with different grades of skin pigmentation within a defined geographical area. Alternatively, the lack of statistically significant differences in CL rates by continent of birth subgroups and the lack of an association with UV light in the recent American data (Newton, 1997) may suggest that these tumours are not caused by excessive sunlight exposure, despite the high incidence reported in Israel.

In summary, a study of CL incidence was carried out using data from multiple sources, based on a defined geographical area with high sunlight exposure. We conclude that CL is apparently more common in Israel than in other countries with available data, but that there is no significant Jewish ethnic variation. Further investigation will be required to confirm whether the high rates for CL are as the result of reporting practices or reflect a true difference in incidence rates. If the latter is the case, aetiological factors, including exposure to sunlight and skin phenotypes, will need to be carefully explored.

### ACKNOWLEDGEMENTS

We are grateful for the contribution of the Israel Cancer Registry staff, particularly Rachel Alon, RA. We thank I Ronen of the Department of Social Medicine at the Hadassah Medical Organization, Jerusalem and Aliza Givon of the Department of Oncology at Rambam Medical Center, Haifa, for their contribution to the abstracting procedure.

### REFERENCES

Bentham G (1996) Association between incidence of non-Hodgkin's lymphoma and solar ultraviolet radiation in England and Wales. *Br Med J* **312**: 1128–1131

Biggar RJ, Horm J, Fraumeni Jr JF, Greene MH and Goedert JJ (1984) Incidence of Kaposi's sarcoma and mycosis fungoides in the United States including Puerto Rico, 1973–81. *J Natl Cancer Inst* **73**: 89–94

Burg G, Dummer R, Dommann S, Nestle F and Nickoloff B (1995) Pathology of cutaneous T-cell lymphoma. In *Cutaneous T-cell lymphoma*, Koh HK and Foss FM. (eds), *Hematology/Oncology Clinics of North America* **9**: 961–995

Cartwright RA, NcNally R and Staines A (1994) The increasing incidence of non-Hodgkin's lymphoma (NHL): the possible role of sunlight. *Leukaemia Lymphoma* **14**: 387–394

Chuang T-Y, Su WPD and Muller SA (1990) Incidence of cutaneous T-cell lymphoma and other rare skin cancers in a defined population. *J Am Acad Dermatol* **23**: 254–256

Coleman MP, Esteve J, Damiecki P, Arslan A and Renard H (1993) *Trends in Cancer Incidence and Mortality.* IARC scientific publ. no. **121**: pp. 641–672. International Agency for Research on Cancer. Lyon

Devesa SS and Fear T (1992) Non-Hodgkin's lymphoma time trends: United States and international data. *Cancer Res* **52** (suppl.): 5432–5440s

Dougan LE, Matthews MLV and Amstrong BK (1981) The effect of diagnostic review on the estimate incidence of lymphatic and hematopoietic neoplasms in Western Australia. *Cancer* **48**: 866–872

Fitzpatrick TB, Pathak MA, Harber LC, Seiji M and Kukita A (1974) *Sunlight and Man.* University of Tokyo Press: Tokyo

Freedman M, Hoar Zahm S and Dosemeci M (1997) Residential and occupational exposure to sunlight and mortality from non-Hodgkin's lymphoma: composite (threefold) case–control study. *Br Med J* **314**: 1451–1455

© Cancer Research Campaign 1998

Hamminga L and van Vloten WA (1980) Report of the Dutch Mycosis Fungoides Study Group. *Br J Dermatol* **102**: 477–478

Hartge P, Devesa SS, Grauman D, Fears TR and Fraumeni JF Jr (1996) Non-Hodgkin's lymphoma and sunlight. *J Natl Cancer Inst* **88**: 298–300

Horn JW, Asire AJ and Young JLC (eds) (1984) *SEER Program: Cancer Incidence and Mortality in the United States 1973–1981*. National Cancer Institutes of Health Publication 85-1837. USA National Cancer Institute: Bethesda

IARC (1992) *Solar and Ultraviolet Radiation*. IARC Monographs on the evaluation of carcinogenic risk to humans. Vol. 55. International Agency for Research on Cancer: Lyon

Iscovich J, Andreev H and Steinitz R (1995) Incidence of cutaneous malignant melanoma in Israel, 1960–1989. *Public Hlth Rev* **23**: 1–23

Kantor AF, Curtis RE and Vondeheid EC (1989) Risk of second malignancies after cutaneous T-cell lymphoma. *Cancer* **63**: 1612–1615

Koh HH, Charif M and Weinstock MA (1995) Epidemiology and clinical manifestations of cutaneous T-cell lymphoma. In *Cutaneous T-cell lymphoma*, Koh HK and Foss FM. (eds), *Hematology/Oncology Clinics of North America* **9**: 943–960

Kricker A, Amstrong BK, Jones ME and Burton RC (eds) (1993) *Health, Solar UV Radiation and Enviromental Change*. IARC Technical Report No. 13. International Agency for Research on Cancer: Lyon

Kripke ML (1990) Effects of UV radiation on tumor immunity. *J Natl Cancer Inst* **82**: 1392–1396

Kripke ML (1994) Ultraviolet radiation and immunology: Something new under the sun. Cancer Res **54**: 6102–6105

McFadden N, Nyfors A, Tanum GC (1983) Mycosis fungoides in Norway 1960–1980: a retrospective study. *Acta Dermatol Venereol* (suppl.) **109**: 1–13

McMichael AJ and Giles GG (1996) Have increases in solar ultraviolet exposure contributed to the rise in incidence of non-Hodgkin's lymphoma? *Br J Cancer* **73**: 945–950

Morrison WL (1989) Effects of ultraviolet radiation on the immune system in humans. *Photochem Photobiol* **50**: 515–524

Myskowski PL (1991) Cutaneous T-cell lymphoma and human immunodeficiency virus. *Arch Dermatol* **127**: 1045–1057

Newton R (1997) Solar ultraviolet radiation is not a major cause of primary cutaneous non-Hodgkin's lymphoma (Letter). *Br Med J* **314**: 1483–1484

Non-Hodgkin's Lymphoma Pathologic Classification Project (1982) National Cancer Institute sponsored study of classification of non-Hodgkin's lymphomas. Summary and description of a working formulation for clinical usage. *Cancer* **49**: 2112–2135

Steinitz R, Parkin DM, Young JL, Bieber CA and Katz L (1989) *Cancer Incidence in Jewish Migrants to Israel 1961–1981*. IARC Scientific Publ. No. 98. International Agency for Research on Cancer: Lyon

Tuyp E, Burgoyne A and Aitchison T (1987) A case–control study of possible causative factors in mycosis fungoides. *Arch Dermatol* **123**: 196–200

Vermeer M, Schmieder GJ, Yoshikawa T, van den Berg JW, Metzman MS, Taylor JR and Strelein JW (1991) Effects of ultraviolet B light on cutaneous immune responses of humans with deeply pigmented skin. *J Invest Dermatol* **97**: 729–734

Weinstock MA (1994) Epidemiology of Mycosis fungoides. *Semin Dermatol* **13**: 154–149

Weinstock MA and Horm JW (1988) Mycosis fungoides in the United States. Increasing incidence and descriptive epidemiology. *JAMA* **260**: 42–46

Whittemore AS, Holly EA and Lee IM (1989) Mycosis fungoides in relation to environmental exposures and immune response: a case–control study. *J Natl Cancer Inst* **181**: 1560–1567

Young JL, Percy CL and Asire AJ (eds) (1981) *Surveillance, Epidemiology and End Results: Incidence and Mortality Data 1973–1977*. NCI Monograph **57**: 1–1082. USA National Cancer Institute: Bethesda

© Cancer Research Campaign 1998